# High prevalence of thyroid hormone autoantibody and low rate of thyroid hormone detection interference

**DOI:** 10.1002/jcla.24124

**Published:** 2021-12-01

**Authors:** Jiajia Ni, Yu Long, Li Zhang, Qingqing Yang, Chunjia Kou, Shuqi Li, Jingyi Li, Haiqing Zhang

**Affiliations:** ^1^ Department of Endocrinology Shandong Provincial Hospital Cheeloo College of Medicine Shandong University Jinan Shandong China; ^2^ Department of Endocrinology People's Hospital of Xiangxi Tujia and Miao Autonomous Prefecture Xiangxi Hunan China; ^3^ Department of Vascular Surgery Shandong Provincial Hospital Shandong First Medical University Jinan Shandong China; ^4^ Department of Endocrinology The first affiliated Hospital of Bengbu Medical College Bengbu Anhui China

**Keywords:** autoimmune thyroid disease, interference, nonthyroid autoimmune diseases, thyroid hormone autoantibody

## Abstract

**Objective:**

Thyroid hormone autoantibody (THAb) is a common antibody in autoimmune disease and can interfere with the detection of thyroid hormone (TH). There was no research reporting the prevalence of THAb in Chinese and the rate of THAb interfering with TH detection.

**Methods:**

We collected 114 patients with autoimmune thyroid disease (AITD) (Hashimoto's thyroiditis, 57 cases; Graves’ disease, 57 cases), 106 patients with nonthyroid autoimmune diseases (NTAID), and 120 healthy subjects. According to the presence or absence of thyroid antibodies, patients with NTAID were divided into two groups: NTAID‐AITD and NTAID groups. Radioimmunoprecipitation technique was used to detect THAb in all subjects. TH was detected on Abbot and Roche platforms in patients with positive THAb.

**Results:**

The prevalence of THAb was 22.8% in Hashimoto's thyroiditis and 45.6% in Graves’ disease. The prevalence of THAb in AITD group was lower than that in NTAID or NTAID‐AITD groups (34.2% vs. 61.5%, *p* = 0.014; 34.2% vs. 71.3%, *p* < 0.01). Among total 98 patients with positive THAb, TH levels of 9 patients were falsely elevated (9.18%).

**Conclusion:**

The prevalence of THAb in AITD patients was lower than that in NTAID patients. Although THAb had a high frequency in various autoimmune diseases, the prevalence of THAb interfering with TH detection was only 9.18%.

## Graphical abstract

We firstly investigated THAb in Chinese patients, and we found the prevalence of THAb in AITD patients was lower than that in NTAID patients. Although THAb has a high frequency in various autoimmune diseases, the rate of THAb interfering with thyroid hormone detection was only 9.18%.

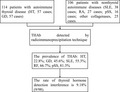

## 1 INTRODUCTION

Thyroid hormone autoantibody (THAb) is an autoantibody binding to thyroid hormone, which is generally believed to be generated by the leakage of iodinated, heterologous thyroglobulin (Tg).^1^ According to categorization based on the thyroid hormones bound and immunoglobulin (Ig) class, THAb has four kinds: IgG antibody against triiodothyronine (T3‐IgG), IgM antibody against triiodothyronine (T3‐IgM), IgG antibody against thyroxine (T4‐IgG), and IgM antibodies against thyroxine (T4‐IgM), in which IgG antibody (IgG‐Ab) is the most common THAb. The prevalence of THAb was approximately 1% in the general population.^2^ A study in Italy revealed that the prevalence of THAb was 20% in Hashimoto's thyroiditis (HT) and 32% in Graves’ disease (GD).^3^ It was believed that the prevalence of THAb in autoimmune thyroid disease (AITD) was lower than that in nonthyroid autoimmune diseases (NTAID).^3^, ^4^, ^5^, ^6^ The mechanism of the presence of THAb in NTAID is still unclear. The above studies were conducted in Italy, and other countries have not carried out any epidemic study on THAb. THAb can bind to thyroid hormone analogs in the detection system, resulting in a falsely elevated level of thyroid hormone. Since THAb was firstly discovered in 1956,^7^ more than ten cases of THAb interfering with thyroid hormone detection have been published.^8^, ^9^, ^10^, ^11^, ^12^, ^13^, ^14^, ^15^, ^16^, ^17^ THAb is a common thyroid autoantibody in autoimmune diseases, but the rate of thyroid hormone detection interference in THAb‐positive patients has not been reported.

A 75‐year‐old woman who suffered from Hashimoto's thyroiditis for 18 years visited our endocrine clinic. Previous results of thyroid function showed that the thyroid‐stimulating hormone (TSH) and free thyroxine (FT4) levels were simultaneously higher than the upper limit of normal value in multiple periods. We suspected that there was interference in FT4 detection, because when we detected FT4 on another platform, the result was significantly different (Roche: 12.7 pmol/L; Abbot: 7.09 pmol/L). THAb is the most common substance interfering with thyroid hormone detection. So, we suspected that THAb interfered with the detection of FT4. However, there was no report on THAb in China, and the normal value of THAb in Chinese was unknown.

Collectively, the current study aimed to establish the reference value of THAb in the Chinese population, determine the prevalence of THAb in Chinese patients with AITD, and evaluate the rate of THAb interfering with thyroid hormone detection in patients with positive THAb.

## 2 PATIENTS AND METHODS

### 2.1 Patients’ selection

We collected 114 patients with AITD who visited in the endocrine clinic of Shandong Provincial Hospital from 2019 to 2020 (57 cases for HT and 57 cases for GD), in which 76 patients were newly diagnosed and 38 patients were under treatment. The exclusion criteria included the following: NTAID, thyroid surgery, and malignancy. We recruited 106 NTAID patients with thyroid dysfunction or thyroid autoantibody positivity who were admitted to the Department of Rheumatology and Immunology, Shandong Provincial Hospital, in which 38 cases were systemic lupus erythematosus (SLE), 27 cases were rheumatoid arthritis (RA), 16 cases were primary Sjogren syndrome (pSS), and 25 cases were other collagenases. Patients who had a history of thyroid surgery or malignancy were excluded. At the same time, we collected 120 healthy subjects who made health examinations in Shandong Provincial Hospital (male: 59 cases, female: 61 cases; mean age (SD): 45.28 (10.36) years). The exclusion criteria included the following: NTAID, thyroid disease, thyroid surgery, and malignancy. The study was approved by the medical ethics committee of Shandong Provincial Hospital, Jinan, Shandong, China.

### 2.2 Diagnosis of autoimmune diseases

AITD included HT and GD. Diagnosis of HT was based on TgAb and/or thyroperoxidase antibody (TPOAb) positivity. GD was defined by thyrotropin receptor antibody (TRAb) positivity and TSH below normal value. The diagnostic standards of SLE and RA were the American Rheumatology Association (ACR) 1997 revised SLE classification criteria^18^ and ACR 1987 revised criteria for the classification of rheumatoid arthritis,^19^ respectively. All pSS patients fulfilled the American‐European Consensus Group 2002 revised classification criteria for pSS.^20^ Other collagenases included connective tissue disease (CTD, 7 cases), vasculitis (7 cases), ankylosing spondylitis (4 cases), osteoarthritis (4 cases), and dermatomyositis (3 cases). Patients were defined as having those collagenases when those collagenases were listed in the diagnoses of discharge letters from hospital. The diagnosis of these collagenases was made by two experienced rheumatologists. NTAID patients were divided into NTAID‐AITD and NTAID groups according to the absence or presence of thyroid antibodies.

### 2.3 THAb assay

THAb was detected by radioimmunoprecipitation technique described in previous researches,^21^ using anti‐human IgM‐Agarose (Sigma‐Aldrich) or protein G (Merck KGAA, Darmstadt, Germany). In brief, 500 μl of serum was incubated with 0.5 μCi ^125^I‐T3 or ^125^I‐T4 (Beijing Fury Runze Biotechnology Co., Ltd) for 60 min at 23 C. Twenty microliters of this mixture was then incubated with 150 μl anti‐human IgM‐Agarose or Protein G, both prediluted 1:10 with saline containing bovine serum albumin (BSA) (Sigma) at a final concentration of 0.5%. After 24‐h incubation at 4℃, tubes were centrifuged at 2,000× g for 1 min and the supernatant was aspirated. Finally, the radioactivity of immunoprecipitation was detected. The percentage of THAb was equal to total radioactivity divided by immunoprecipitation radioactivity.

### 2.4 Thyroid function and autoantibody measurements

Thyroid function and autoantibodies were detected by electrochemiluminescence (Roche, Cobas e601). The reference ranges for FT4, free triiodothyronine (FT3), TSH, TgAb, and TPOAb are 12.0−22.0 pmol/L, 3.1−6.8 pmol/L, 0.27−4.20 µIU/ml, 0−115 IU/ml, and 0−34 IU/ml, respectively. We used the Architect immunoassay (Abbot Diagnostics) to re‐evaluate FT3 and FT4 for patients with positive THAb. The reference ranges for FT4 and FT3 in Abbot are 9.01−19.05 pmol/L, 2.43−6.01 pmol/L.

### 2.5 Statistical analysis

Continuous variables were expressed as the mean (M) ± standard deviation (SD). The Kolmogorov‐Smirnov test was used to examine whether the data of THAb in the healthy obeyed Gaussian distribution. Categorical variables were expressed by percentages. Categorical variables were compared by the chi‐squared test and Fisher tests. Continuous variables were compared by t tests and Kruskal‐Wallis test. SPSS version 25 was applied in statistical analysis. *p* < 0.05 indicated that the difference was statistically significant.

## 3 RESULTS

### 3.1 Normal value

T3‐IgG, T3‐IgM, T4‐IgG, and T4‐IgM in the healthy obeyed Gaussian distribution. We used M + 2.56SD to calculate the normal value of four types of THAb: T3‐IgG, 0–4.45%; T3‐IgM, 0–4.62%; T4‐IgG, 0–3.98%; and T4‐IgM, 0–4.20%.

### 3.2 Demographic characteristics and the prevalence of THAb in AITD, NTAID, and NTAID‐AITD groups

There was no significant difference in the proportion of females among the three groups. The mean age of the NTAID‐AITD group was higher than that of the AITD group (47.77 ± 14.80 years vs. 38.82 ± 12.25 years, *p *= 0.002). There was no significant difference in mean age between the NTAID‐AITD group and the NTAID group or between the AITD group and the NTAID group. The prevalence of THAb in AITD group was lower than that in NTAID or NTAID‐AITD groups (34.2% vs. 61.5%, *p *= 0.014; 34.2% vs. 71.3%, *p *< 0.01). As to T3‐IgG, there was no significant difference among the three groups. AITD group had a lower frequency of T3‐IgM, T4‐IgG, and T4‐IgM than NTAID‐AITD group (12.3% vs. 36.3%, *p *< 0.001; 14.9% vs. 35.0%, *p *= 0.002; 4.4% vs. 36.3%, *p *< 0.001). Both NTAID and NTAID‐AITD groups had higher prevalence of T4‐IgM than AITD group (38.5% vs. 4.4%, *p *< 0.001; 36.3% vs. 4.4%, *p *< 0.001). With the classification base on specificity thyroid hormone bound, both NTAID‐AITD and NTAID groups had a higher prevalence of T3‐T4Ab than AITD group (23.8% vs. 6.1%, *p *= 0.001; 23.1% vs. 6.1%, *p *= 0.016). As to classification based on Ig type, both NTAID‐AITD and NTAID groups had a higher prevalence of IgG and IgM‐Ab than the AITD group (32.5% vs. 7.9%, *p *< 0.001; 34.6% vs. 7.9%, *p *= 0.001). NTAID‐AITD group also had a higher prevalence of isolated IgM‐Ab than the AITD group (26.3% vs. 8.8%, *p *= 0.002). There was no significant difference in the distribution of THAb between NTAID‐AITD and NTAID groups (Table 1).

**TABLE 1:** Demographic characteristic and the prevalence of different kinds of THAb in AITD, NTAID, and NTAID‐AITD groups

	AITD	NTAID	NTAID‐AITD	*p*
Number	114	26	80	
Female, n (%)	96 (84.2)	24 (92.3)	73 (87.7)	0.246
Age, y, M ± SD	38.8 ± 12.3^a^	46.6 ± 16.9	47.8 ± 14.8^b^	**<0.001**
THAb, n (%)	39 (34.2)^a^	16 (61.5)^b^	57 (71.3)^b^	**<0.001**
T3‐IgG, n (%)	17 (14.9)	6 (23.1)	14 (17.5)	0.607
T3‐IgM, n (%)	14 (12.3)^a^	7 (26.9)	29 (36.3)^b^	**<0.001**
T4‐IgG, n (%)	17 (14.9)^a^	8 (30.8)	28 (35.0)^b^	**0.004**
T4‐IgM, n (%)	5 (4.4)^a^	10 (38.5)^b^	29 (36.3)^b^	**<0.001**
Only T3Ab, n (%)	18 (15.8)	5 (19.2)	18 (22.5)	0.499
Only T4Ab, n (%)	14 (12.3)	5 (19.2)	20 (25.0)	0.073
T3‐T4Ab, n (%)	7 (6.1)^a^	6 (23.1)^b^	19 (23.8)^b^	**0.001**
Only IgG‐Ab, n (%)	20 (17.5)	2 (7.7)	10 (12.5)	0.330
Only IgM‐Ab, n (%)	10 (8.8)^a^	5 (19.2)	21 (26.3)^b^	**0.005**
IgG and IgM‐Ab, n (%)	9 (7.9)^a^	9 (34.6)^b^	26 (32.5)^b^	**<0.001**

^a, b:^ The different letters of the superscript indicated that there were statistical differences between groups, *p* < 0.05.

Bold values are statistical differences *p* < 0.05.

### 3.3 Distribution of different kinds of THAb in HT, GD, SLE, RF, pSS

**FIGURE 1:**
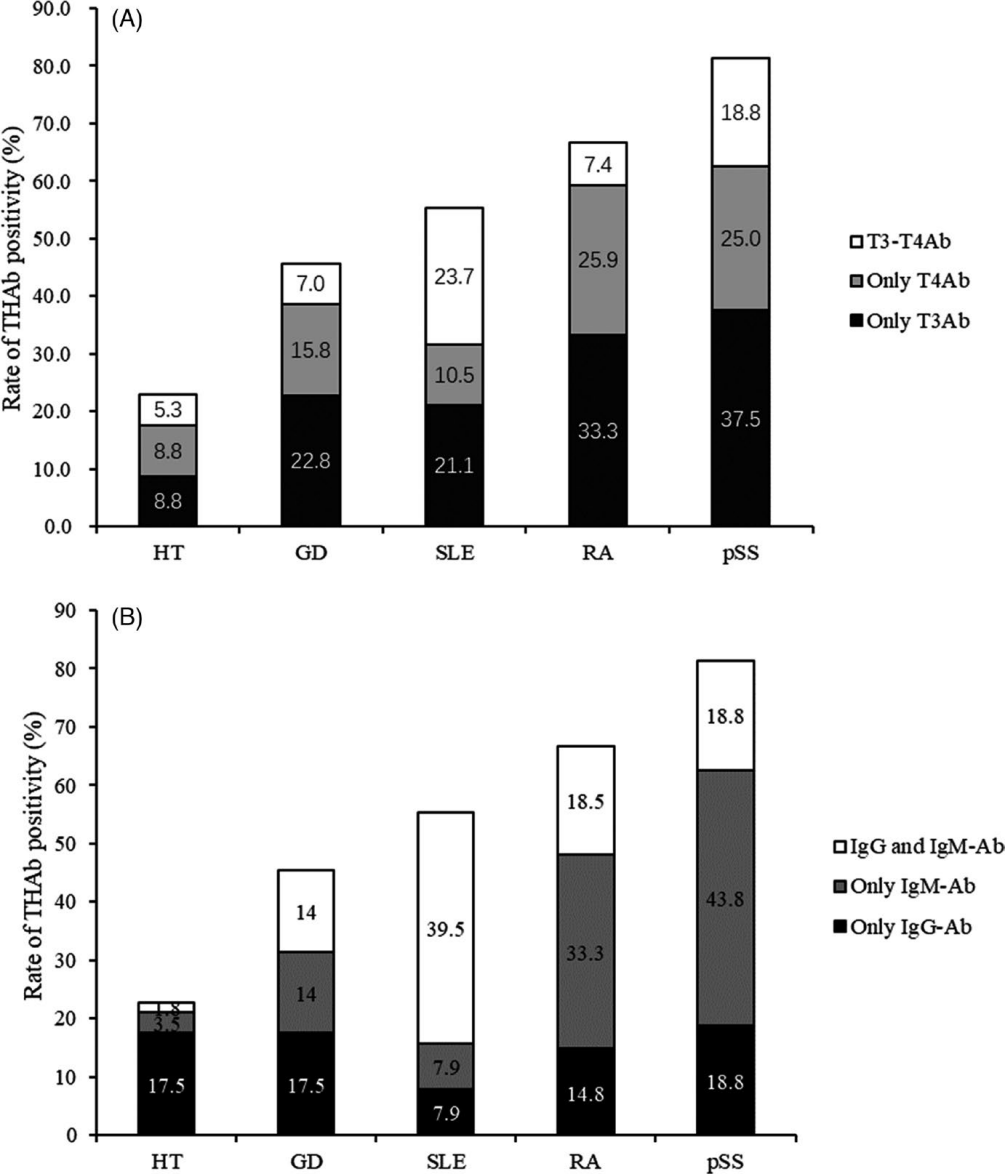
Distribution of different kinds of THAb (A: classification based on hormones bound; B: classification based on Ig class) in five disease groups: HT, GD, SLE, RA, and pSS

The overall prevalence of THAb in HT was lower than that in SLE, RF, pSS (22.8% vs. 55.3%, *p *= 0.003; 22.8% vs. 66.7%, *p *< 0.001; 22.8% vs. 81.3%, *p *< 0.001). Both RA and pSS groups had a higher prevalence of isolated T3Ab than HT group (*p *= 0.010; *p *= 0.015). RA group had a higher prevalence of isolated T4Ab than HT group (*p *= 0.048). The prevalence of T3‐T4Ab was higher in SLE than that in HT (*p *= 0.012). RA and pSS groups had a higher prevalence of isolated IgM‐Ab than HT group (*p *< 0.001; *p *< 0.001). SLE group had a higher prevalence of IgG and IgM‐Ab than HT group (*p *< 0.001). GD group had a lower prevalence of T3‐T4Ab and IgG and IgM‐Ab than SLE group (*p *= 0.044; *p *= 0.010) and had a lower prevalence of overall THAb and isolated IgM‐Ab than pSS group ((*p *= 0.025; *p *= 0.016) (Figure 1).

### 3.4 Interference in thyroid hormone detection

A total of 98 patients with THAb positive re‐detected FT3 and FT4 on the Abbott platform, and the results of thyroid hormones on two platforms were inconsistent in 9 patients (9.18%). One patient had interference in FT3 detection, and 8 patients had interference in FT4 detection. The patient with interference in FT3 detection had T4‐IgG. In patients with falsely elevated FT4, 3 patients had T4Ab, 4 patients had T3Ab, and 1 patient had T3‐T4Ab. There was no difference in the prevalence of thyroid hormone detection interference among different diseases (*p *=  0.667) (Table 2).

**TABLE 2:** Nine patients with falsely elevated thyroid hormone levels

	FT3**^c^** pmol/l	FT3**^d^** pmol/l	FT4**^c^** pmol/l	FT4**^d^** pmol/l	TSH**^c^** µIU/ml	T3‐IgG	T3‐IgM	T4‐IgG	T4‐IgM	Disease
1	3.26	3.15	13.21	**8.98**	4.067	**‐**	**‐**	**‐**	+	CTD
2	3.88	3.18	**28.44**	17.49	<0.005	**‐**	+	+	+	pSS
3	4.02	**1.79**	17.80	11.38	2.700	‐	**‐**	+	**‐**	RA
4	3.52	3.01	**23.9**	18.38	0.081	+	**‐**	**‐**	**‐**	SLE
5	1.42	<1.46	12.4	**8.77**	0.248	+	+	**‐**	**‐**	SLE
6	23.8	6.17	**53.1**	16.83	<0.005	**‐**	+	**‐**	**‐**	GD
7	5.7	4.17	**22.1**	15.81	<0.005	**‐**	**‐**	+	**‐**	GD
8	6.47	4.01	**24.6**	10.5	<0.005	**‐**	+	**‐**	**‐**	HT
9	5.09	4.66	**24.5**	16.02	0.045	**‐**	**‐**	+	**‐**	HT

^c:^ Thyroid hormones and TSH were detected on Roche platform.

^d:^ Thyroid hormones were detected on Abbot platform.

Bold values are statistical differences *p* < 0.05.

## 4 DISCUSSION

In our research, we calculated the normal value of THAb: 0–4.45% (T3‐IgG), 0–4.62% (T3‐IgM), 0–3.98% (T4‐IgG), and 0–4.20% (T4‐IgM) and determined that the prevalence of THAb was 22.8% in HT and 45.6% in GD. In 1997, Benvenga et al. firstly used radioimmunoprecipitation technique to detect THAb and obtained the normal value of THAb: 0–3.6% (T3‐IgG), 0–3.9% (T3‐IgM), 0–3.9% (T4‐IgG), and 0–3.4% (T4‐IgM).^1^ Subsequently, in 2002, Benvenga et al. reported that the positive rate of THAb was 20% in HT and 32% in GD in Italy.^3^ Both the normal value and prevalence of THAb in our study were slightly higher than those in Benvenga's study. The following two reasons might account for this inconsistency: (a) Genetic and environmental differences driven by geographical differences. (b) Different reagents used in experiments had different specificities and affinities. Due to limited conditions, we could not get the same reagents as that in Benvenga's studies. We used protein G and anti‐human IgM‐agarose instead of anti‐human IgG/ IgM serum.

Although THAb can bind to thyroid hormone, thyroid hormone is a hapten and cannot directly elicit the production of antibody. In 1972, Yukio et al. reported that rabbits immunized with mild denatured thyroglobulin (Tg) produced antibodies that could bind to thyroid hormone in the gamma‐globulin region.^22^ In 1997, Benvenga found that iodinated, heterologous thyroglobulin could lead to the production of THAb in patients with FNAB, and THAb had a positive relationship with TgAb.^1^ In this research, Benvenga also demonstrated that the prevalence of THAb in HT was 10‐fold higher than that in nonautoimmune thyroid diseases. According to the aftermentioned studies, it was generally believed that THAb was generated due to the leakage of Tg and was the subtype of TgAb. However, in our study, there was no difference in the prevalence of THAb between NTAID and NTAID‐AITD groups. Meanwhile, the distribution of THAb in NTAID and AITD groups was significantly different. AITD patients had a higher proportion of single THAb, while NTAID patients had a higher proportion of composite THAb (T3‐T4Ab, IgG, and IgM‐Ab). Previous studies have reported that the prevalence of THAb in NTAID was much higher than that in AITD. The prevalence of THAb is 50% in SS, 26% in RA,^3^ 92.3%^5^ in type 1 diabetes (DM1), and 97% in vitiligo.^4^ Most of these patients were euthyroid and thyroid autoantibody negative. Researches on patients with FNAB^1^ and patients receiving tyrosine kinase inhibitor treatment^23^ indicated that THAb was the earliest thyroid autoantibody occurring in early thyroid damage. Furthermore, a study reported that RA patients with positive THAb would develop to autoimmune hypothyroidism in the following 10  years.^3^ The above studies implied that the production of THAb required the damage of the thyroid rather than the presence of TgAb. Collectively, we speculated that the mechanism of the production of THAb in AITD and NTAID was different. In NTAID, thyroid damage comes from systemic immune injury rather than AITD. In NTAID, immune complexes can deposit in the vessel wall of the thyroid. Complement activation will cause vascular occlusion, which will lead to insufficient oxygen supply in thyroid tissue and eventually result in thyroid injury. Research on THAb and DM1 found that THAb had a relationship with microangiopathy.^5^ Immune complexes depositing in the thyroid lead to immune cell infiltration (T and B lymphocytes) and cytokine release (INF‐γ, IL‐6, IL‐12, IL‐21, and IL‐17), like Lupus nephritis,^24^ which will exacerbate thyroid tissue damage.

THAb can interfere with thyroid hormone detection, which may cause laboratory results inconsistent with clinical manifestations, and lead to misdiagnosis of clinicians. Since THAb was first discovered in 1956,^7^ more than ten cases of THAb interfering with thyroid hormone detection have been published.^8^, ^9^, ^10^, ^11^, ^12^, ^13^, ^14^, ^15^, ^16^, ^17^ In a one‐step immunoassay (like Roche), labeled thyroid hormone analog and patient's serum were added to the reaction system at the same time to compete for the solid‐phase antibody. The uncombined material was then flushed away, with only the combined thyroid hormone analog measured. THAb could bind to the labeled thyroid hormone analogs, which resulted in a decrease in the labeled thyroid hormone analogs bound to the solid‐phase antibody and falsely elevated thyroid hormone value. In a two‐step assay (like Abbot), uncombined material was washed away before adding the patient's serum. Therefore, there was no contact between the patient's serum and the labeled thyroid hormone analog. In our study, among 98 patients with positive THAb, only 9 patients (9.18%) had inconsistent results between Roche and Abbott platforms. Thyroid hormone analogs used in experimental and commercial platforms were different, which might explain the low interference rate of THAb. Titer and affinity of THAb might also affect the occurrence of detection interference. Although the prevalence of immunoassay interference was less than 1/10, THAb had a huge impact on some diseases that had a high THAB‐positive rate (DM1, vitiligo). When the clinical manifestations of patients were not consistent with the results of laboratory tests, clinicians should consider whether there was THAb interfering with detection. Among the 9 patients whose thyroid hormone detection was interfered, 8 patients’ FT4 detections were interfered. And the type of falsely elevated thyroid hormones did not correspond to the type of THAb (Table 2). The case mentioned in the introduction had T3‐IgM and T4‐IgG, but only FT4 detection was interfered. This phenomenon might arise from 90% of thyroid hormone in serum was thyroxine (T4). At the same time, we did not find a linear relationship between the titer of THAb and the hormone detection difference between the two platforms. So, the affinity and functional domains of THAb need to be further researched.

There were some strengths of this study. It was the first research that investigated THAb in the Chinese population. We selected 120 healthy subjects with a sex ratio of one to one and used a 99% reference interval to establish normal values, which made our reference values more credible. The normal value of THAb established by us could provide a reference for future research on THAb in China. The prevalence of THAb in SLE was described for the first time. Furthermore, we determined the rate of THAb interfering with thyroid hormone detection. However, this study also had some limitations. The sample size of patients in this study was small, and there might be selection bias. On the other hand, the use of glucocorticoids and immunosuppressants (about 56.6% of NTAID patients had a history of glucocorticoid or immunosuppressant use in recent 6 months, but the duration and dose of glucocorticoid use were unknown) was not collected.

## 5 CONCLUSION

The prevalence of THAb in the general population was lower than that in AITD patients, and the prevalence of THAb in AITD patients was lower than that in NTAID patients. Although THAb has a high frequency in various autoimmune diseases, the rate of THAb interfering with thyroid hormone detection was only 9.18%.

## Data Availability

Research data are not shared.

## References

[1] Benvenga S , Bartolone L , Squadrito S , Trimarchi F . Thyroid hormone autoantibodies elicited by diagnostic fine needle biopsy. J Clin Endocrinol Metab. 1997;82(12):4217‐4223. 9398743 10.1210/jcem.82.12.4420

[2] Benvenga S , Burek CL , Talor M , Rose NR , Trimarchi F . Heterogeneity of the thyroglobulin epitopes associated with circulating thyroid hormone autoantibodies in Hashimoto's thyroiditis and non‐autoimmune thyroid diseases. J Endocrinol Invest. 2002;25(11):977‐982. 12553558 10.1007/BF03344071

[3] Ruggeri RM , Galletti M , Mandolfino MG , et al. Thyroid hormone autoantibodies in primary Sjögren syndrome and rheumatoid arthritis are more prevalent than in autoimmune thyroid disease, becoming progressively more frequent in these diseases. J Endocrinol Invest. 2002;25(5):447‐454. 12035942 10.1007/BF03344036

[4] Colucci R , Lotti F , Dragoni F , et al. High prevalence of circulating autoantibodies against thyroid hormones in vitiligo and correlation with clinical and historical parameters of patients. Br J Dermatol. 2014;171(4):786‐798. 25059078 10.1111/bjd.13286

[5] Benvenga S , Pintaudi B , Vita R , Di Vieste G , Di Benedetto A . Serum thyroid hormone autoantibodies in type 1 diabetes mellitus. J Clin Endocrinol Metab. 2015;100(5):1870‐1878. 25710564 10.1210/jc.2014-3950

[6] Vita R , Santaguida MG , Virili C , et al. Serum thyroid hormone antibodies are frequent in patients with polyglandular autoimmune syndrome type 3, particularly in those who require thyroxine treatment. Front Endocrinol (Lausanne). 2017;8:212. 28894436 10.3389/fendo.2017.00212 PMC5581384

[7] Robbins J , Rall JE , Rawson RW . An unusual instance of thyroxine‐binding by human serum gamma globulin. J Clin Endocrinol Metab. 1956;16(5):573‐579. 13319450 10.1210/jcem-16-5-573

[8] Lewandowski KC , Dąbrowska K , Lewiński A . Case report: when measured free T4 and free T3 may be misleading. Interference with free thyroid hormones measurements on Roche^®^ and Siemens^®^ platforms. Thyroid Res. 2012;5(1):11. 23107155 10.1186/1756-6614-5-11 PMC3520776

[9] Lee MN , Lee SY , Hur KY , Park HD . Thyroxine (T4) autoantibody interference of free T4 concentration measurement in a patient with hashimoto's thyroiditis. Ann Lab Med. 2017;37(2):169‐171. 28029007 10.3343/alm.2017.37.2.169 PMC5203998

[10] Teti C , Nazzari E , Galletti MR , et al. Unexpected elevated free thyroid hormones in pregnancy. Thyroid. 2016;26(11):1640‐1644. 27538922 10.1089/thy.2016.0112

[11] Wu SY , Green WL . Triiodothyronine (T3)‐binding immunoglobulins in a euthyroid woman: effects on measurement of T3 (RIA) and on T3 turnover. J Clin Endocrinol Metab. 1976;42(4):642‐652. 1262440 10.1210/jcem-42-4-642

[12] Ginsberg J , Segal D , Ehrlich RM , Walfish PG . Inappropriate triiodothyronine (T3) and thyroxine (T4) radioimmunoassay levels secondary to circulating thyroid hormone autoantibodies. Clin Endocrinol (Oxf). 1978;8(2):133‐139. 580230 10.1111/j.1365-2265.1978.tb02161.x

[13] Nakamura S , Sakata S , Komaki T , Kamikubo K , Yasuda K , Miura K . An improved and simplified method for the detection of thyroid hormone autoantibodies (THAA) in serum. Endocrinol Jpn. 1986;33(3):415‐422. 3757925 10.1507/endocrj1954.33.415

[14] Abs R , Martin M , Blockx P . Changes in serum thyroid hormone autoantibody concentrations during pregnancy: a case report. Horm Res. 1991;35(5):205‐207. 1802824 10.1159/000181903

[15] Srichomkwun P , Scherberg NH , Jakšić J , Refetoff S . Diagnostic dilemma in discordant thyroid function tests due to thyroid hormone autoantibodies. AACE Clin Case Rep. 2017;3(1):e22‐e25. 28078322 10.4158/EP151142.CR PMC5222615

[16] Ohshiro K , Sakata S , Matsuda M , et al. A case of hypothyroidism with simultaneous presence of stimulating type anti‐thyrotropin (TSH) receptor antibodies and anti‐thyroxine (T4) autoantibodies. Endocrinol Jpn. 1992;39(3):245‐250. 1425450 10.1507/endocrj1954.39.245

[17] Shimon I , Pariente C , Shlomo‐David J , Grossman Z , Sack J . Transient elevation of triiodothyronine caused by triiodothyronine autoantibody associated with acute Epstein‐Barr‐virus infection. Thyroid. 2003;13(2):211‐215. 12699597 10.1089/105072503321319530

[18] Hochberg MC . Updating the American College of Rheumatology revised criteria for the classification of systemic lupus erythematosus. Arthritis Rheum. 1997;40(9):1725. 10.1002/art.1780400928 9324032

[19] Arnett FC , Edworthy SM , Bloch DA , et al. The American Rheumatism Association 1987 revised criteria for the classification of rheumatoid arthritis. Arthritis Rheum. 1988;31(3):315‐324. 3358796 10.1002/art.1780310302

[20] Vitali C , Bombardieri S , Jonsson R , et al. Classification criteria for Sjögren's syndrome: a revised version of the European criteria proposed by the American‐European Consensus Group. Ann Rheum Dis. 2002;61(6):554‐558. 12006334 10.1136/ard.61.6.554 PMC1754137

[21] Benvenga S , Vita R , Di Bari F , et al. Assessment of serum thyroid hormone autoantibodies in the first trimester of gestation as predictors of postpartum thyroiditis. J Clin Transl Endocrinol. 2019;18:100201. 31428563 10.1016/j.jcte.2019.100201 PMC6693681

[22] Ochi Y , Shiomi K , Hachiya T , Yoshimura M , Miyazaki T . Immunological analysis of abnormal binding of thyroid hormone in the gamma globulin. J Clin Endocrinol Metab. 1972;35(5):743‐752. 4116067 10.1210/jcem-35-5-743

[23] Mondello P , Mian M , Pitini V , et al. Thyroid hormone autoantibodies: are they a better marker to detect early thyroid damage in patients with hematologic cancers receiving tyrosine kinase inhibitor or immunoregulatory drug treatments? Curr Oncol. 2016;23(3):e165‐e170. 27330353 10.3747/co.23.3026 PMC4900836

[24] Anders HJ , Saxena R , Zhao MH , Parodis I , Salmon JE , Mohan C . Lupus Nephritis. Nat Rev Dis Primers. 2020;6(1):7. 31974366 10.1038/s41572-019-0141-9

